# Interferon regulatory factor 5 (*IRF5*) gene variants are associated with multiple sclerosis in three distinct populations

**DOI:** 10.1136/jmg.2007.055012

**Published:** 2008-02-19

**Authors:** G Kristjansdottir, J K Sandling, A Bonetti, I M Roos, L Milani, C Wang, S M Gustafsdottir, S Sigurdsson, A Lundmark, P J Tienari, K Koivisto, I Elovaara, T Pirttilä, M Reunanen, L Peltonen, J Saarela, J Hillert, T Olsson, U Landegren, A Alcina, O Fernández, L Leyva, M Guerrero, M Lucas, G Izquierdo, F Matesanz, A-C Syvänen

**Affiliations:** 1 Molecular Medicine, Department of Medical Sciences, Uppsala University, Uppsala, Sweden; 2 Department of Neurology, Helsinki University Central Hospital and Molecular Neurology Research Program, University of Helsinki, Helsinki, Finland; 3 Department of Clinical Neuroscience, Division of Neurology, Karolinska Institutet, Karolinska University Hospital Huddinge, Stockholm, Sweden; 4 Rudbeck Laboratory, Department of Genetics and Pathology, Uppsala University, Uppsala, Sweden; 5 Central Hospital of Seinäjoki, Seinäjoki, Finland; 6 Department of Neurology, University of Tampere and Tampere University Hospital, Tampere, Finland; 7 Department of Neurology and Neuroscience, University of Kuopio and Kuopio University Hospital, Kuopio, Finland; 8 Department Neurology, University of Oulu and Oulu University Hospital, Oulu, Finland; 9 Department of Molecular Medicine, Biomedicum, National Public Health Institute, Helsinki, Finland; 10 Department of Clinical Neuroscience, Neuroimmunology Unit, Karolinska Institutet, Stockholm, Sweden; 11 Instituto de Parasitología y Biomedicina López Neyra, Consejo Superior de Investigaciones Científicas, Granada, Spain; 12 Servicio de Neurología, Instituto de Neurociencias Clínicas, Hospital Regional, Universitario Carlos Haya, Málaga, Spain; 13 Servicio de Neurología, Hospital Clínico San Cecilio, Granada, Spain; 14 Servicio de Biología Moleular, Hospital Universitario Virgen Macarena, Sevilla, Spain; 15 Unidad de Esclerosis Múltiple, Hospital Universitario Virgen Macarena, Sevilla, Spain

## Abstract

**Background:**

*IRF5* is a transcription factor involved both in the type I interferon and the toll-like receptor signalling pathways. Previously, *IRF5* has been found to be associated with systemic lupus erythematosus, rheumatoid arthritis and inflammatory bowel diseases. Here we investigated whether polymorphisms in the *IRF5* gene would be associated with yet another disease with features of autoimmunity, multiple sclerosis (MS).

**Methods:**

We genotyped nine single nucleotide polymorphisms and one insertion-deletion polymorphism in the *IRF5* gene in a collection of 2337 patients with MS and 2813 controls from three populations: two case–control cohorts from Spain and Sweden, and a set of MS trio families from Finland.

**Results:**

Two single nucleotide polymorphism (SNPs) (rs4728142, rs3807306), and a 5 bp insertion-deletion polymorphism located in the promoter and first intron of the *IRF5* gene, showed association signals with values of p<0.001 when the data from all cohorts were combined. The predisposing alleles were present on the same common haplotype in all populations. Using electrophoretic mobility shift assays we observed allele specific differences in protein binding for the SNP rs4728142 and the 5 bp indel, and by a proximity ligation assay we demonstrated increased binding of the transcription factor SP1 to the risk allele of the 5 bp indel.

**Conclusion:**

These findings add *IRF5* to the short list of genes shown to be associated with MS in more than one population. Our study adds to the evidence that there might be genes or pathways that are common in multiple autoimmune diseases, and that the type I interferon system is likely to be involved in the development of these diseases.

Multiple sclerosis (MS, OMIM 126200) is an inflammatory disease estimated to affect over two million individuals worldwide. MS is not well recognised as an autoimmune disease, but exhibits features of autoimmunity—that is, activation of the immune system in the absence of apparent ongoing infection.^1^ In MS the presumed target for the autoimmune process is the central nervous system, and the disease manifests itself by immune mediated demyelination and damage to axons. A spectrum of neurological symptoms are found among MS patients, including sensory or motor pareses, visual disturbances, ataxia, pain, cognitive dysfunction and fatigue. MS is a complex disease caused by interaction between environmental and inherited factors. The disease shows familial clustering, and twin studies have revealed that a large portion of this clustering can be attributed to shared genes.^2^

A remarkably low number of susceptibility genes for MS have been identified so far. Genome-wide linkage studies have indicated several possible susceptibility loci, but the only locus to be identified across most studies is the major histocompatibility complex (MHC) on chromosome 6p21, were the HLA-DRB1*1501 allele is a well established genetic risk factor for MS.^3^ ^4^ This locus does not, however, account for the whole genetic component of MS, and multiple loci with smaller contributions to disease susceptibility are likely to exist. Numerous candidate gene studies have also been performed in MS, but findings from one population have been difficult to replicate in other populations. The protein kinase C α (PRKCA) gene is one of few genes reported to be associated with MS in more than one population.^5^ ^6^ Recently, the interleukin 7 receptor α chain gene (IL7RA) was found to be associated with MS in two independent candidate gene studies and in a genome-wide association study.^7^^–^9 The genome-wide association study also identified variants in the interleukin 2 receptor α chain gene (IL2RA) as risk factors for MS,^7^ which is in accordance with findings in a previous candidate gene study.^10^

It is relatively common that patients affected by an autoimmune disease suffer from another autoimmune disease, and that members of the same family suffer from different autoimmune diseases. For example, in families with systemic lupus erythematosus (SLE, OMIM 152700), MS and rheumatoid arthritis (RA, OMIM 180300) occur more frequently than in the general population.^11^ Such observations suggest shared genes or involvement of common cellular pathways in these diseases. This hypothesis is supported by reports on genes found to be associated with more than one autoimmune disease in experimental models of RA and MS.^12^ Shared genes in autoimmune diseases are becoming apparent also in humans, such as *PTPN22* in RA and SLE,^13^ *MHC2TA* in RA and MS,^14^ and recently the suggested involvement of *CD24* in MS and SLE.^15^

The type I interferon (IFN) system has been postulated to play a key role in autoimmune diseases.^16^ Increased expression of IFN induced genes has been detected in autoimmune diseases like SLE,^17^ RA,^18^ Sjögren’s syndrome,^19^ and in a subgroup of MS patients.^20^ The interferon regulatory factors (IRFs) are major regulators of genes activated by the type I IFNs,^21^ and a role in the regulation of the immune system is well established for the majority of the members of the IRF family of nine genes. The role of *IRF5* in the immune response is not as well established as for other IRFs, but *IRF5* has recently received attention in studies on autoimmunity. The *IRF5* gene displays a complex transcription pattern with three alternative non-coding 5′ exons and at least nine alternatively spliced mRNAs.^22^ *IRF5* is expressed in dendritic cells, monocytes and B cells, but its expression can be induced in other cell types by the type I IFNs.^23^ *IRF5* regulates the toll-like receptor (TLR) dependent activation of inflammatory cytokines and functions downstream of the TLR-MyD88 pathway where it is activated by MyD88 and TNF receptor associated factor 6 (TRAF6).^24^ Our original finding of an association between the *IRF5* gene and SLE,^25^ which has been replicated in multiple populations, as well as our recent findings of association between *IRF5* and RA^26^ and inflammatory bowel diseases (IBD),^27^ provide additional support for the important role of *IRF5* and the type I IFN system in autoimmune diseases. Inspired by these findings, and by the role of recombinant IFN β as a standard treatment of MS, we investigated whether polymorphisms in the *IRF5* gene would also be associated with MS. We found that polymorphisms in the *IRF5* gene displayed associations with MS in three independent patient cohorts from Spain, Sweden and Finland. The three most strongly associated polymorphisms are located in the promoter region and first intron of *IRF5*. A functional role is suggested by increased protein binding to the risk alleles of two of these polymorphisms.

## PATIENTS AND METHODS

### Clinical samples

Cohorts of MS patients collected in Spain, Sweden and Finland were included in the study. All patients had clinically or laboratory supported definite MS according to the Poser criteria^28^ or fulfilled the criteria of McDonald for MS.^29^ In total 660 Spanish MS patients were recruited at three public hospitals in the south of Spain: Clínico in Granada (n = 130), Carlos Haya in Málaga (n = 363) and Virgen de la Macarena in Seville (n = 167). The Spanish controls were 833 blood donors without history of inflammatory disease attending the blood banks of Granada (n = 441), Seville (n = 211) and Málaga (n = 181); 67% of the patients and 50% of the controls were female. The Swedish cohort consisted of 1166 MS patients (67% females) and 1235 controls (63% females) of Nordic ethnicity recruited at Danderyd’s Hospital or Karolinska University Hospital in Huddinge or in Solna, all located in the Stockholm County of Sweden. Controls were consecutive blood donors of Nordic origin that visited three blood donor facilities in the Stockholm area in 2001 and in 2004/2005. The Finnish cohort consisted of 511 MS trio-families recruited at five centres in Finland: the University Central Hospitals in Helsinki (n = 148 families), Tampere (n = 102), Kuopio (n = 79), Oulu (n = 73), and the Central Hospital of Seinäjoki (n = 109); 71% of the Finnish patients were female. The study was approved by the respective local ethics committees and informed consent was obtained from all study participants.

### Genotyping

Nine single nucleotide polymorphisms (SNPs) and one 5 bp bi-alleic insertion-deletion polymorphism (CGGGG indel) in the *IRF5* gene were genotyped. The SNPs were genotyped using multiplex fluorescent minisequencing (single base extension) with the SNPstream system (Beckman Coulter).^30^ The SNP rs4728142 was also genotyped using a homogeneous minisequencing assay with fluorescence polarisation detection (FP-TDI) (Analyst AD, Molecular Probes) in 1440 Swedish samples. The genotype call rate in the samples was on average 96.6% and the genotyping accuracy was 99.8% as estimated from 13.800 genotype comparisons (20% of the genotypes) between repeated assays. The CGGGG indel was amplified as a 100/105 bp polymerase chain reaction (PCR) fragment using fluorescent primers with subsequent fragment analysis on an ABI PRISM 3730 DNA Analyzer (Applied Biosystems, Foster City, California, USA). The GeneMapper v.3.7. software was used for genotype calling. Alternatively, the PCR amplified fragments were separated on 4% MetaPhor high resolution agarose gels (Cambrex Bio Science Rockland Inc, Maine, USA) and visualised using ethidium bromide staining. The call rate for genotyping the CGGGG indel was on average 96.0% in the three cohorts and the genotyping accuracy was 99.1% as estimated from genotype comparison between repeated assays for 15% of the subjects. Samples with a genotype call rate <80% for the 10 markers and four trios with inheritance errors were excluded from the analysis. The PCR and extension primers are provided in the supplementary table S1. All genotyped polymorphisms fulfilled the criteria of Hardy–Weinberg equilibrium in the control samples.

### Electrophoretic mobility shift assay (EMSA)

For each allele of the polymorphism, pairs of single stranded 5′ biotinylated and unlabelled 30–37 bp oligonucleotides (obtained from IDT Inc, Coralville, Iowa, USA) were allowed to anneal to generate double stranded probes (supplementary table S2). Twenty fmoles of labelled probes was incubated for 20 min with 2 μl of nuclear extract prepared from blood cells in a freshly made binding buffer supplemented with poly(dI-dC)ṡpoly(dI-dC) and protease inhibitors. Competition experiments were performed using a 100-fold molar excess of unlabelled probe. The binding reactions were analysed using electrophoresis on 6% polyacrylamide gels and transferred to nylon membranes (Bio-Rad Laboratories, Hercules, California, USA). The biotinylated fragments were detected by a chemiluminescent procedure (LightShift Chemiluminescent EMSA kit, Pierce Biotechnology, Rockford, Illinois, USA).

### Proximity ligation assay (PLA)

Polyclonal antibody against SP1 was purchased from Santa Cruz Biotechnology (Cat. no. sc-14027, Santa Cruz, California, USA). The antibody was biotinylated using D-biotin-*N*-hydroxysuccinimide (Nordic Biosite, Täby, Sweden) according to the recommendation by the manufacturer. The biotinylated antibody was diluted in phosphate buffered saline (PBS) containing 1% bovine serum albumin (BSA) to a final concentration of 100 nM.  The anti-SP1 antibody was then combined in a 1:1 ratio with a streptavidin–oligonucleotide conjugate (SoluLink, San Diego, California, USA) in a volume of 20 μl, incubated at room temperature for 1 h, and then stored at 4°C until use. High pressure liquid chromatography (HPLC) purified DNA probes (Biomers.net, Ulm, Germany) were made partially double stranded according to Gustafsdottir *et al*.^31^ The partially double stranded DNA probes (25 pM) were incubated with 200 ng of Jurkat nuclear extract (Active Motif, Carlsbad, California, USA) in PBS containing 1% BSA, 16 μg/ml of sheared polyA bulk nucleic acid (Sigma-Aldrich, Stockholm, Sweden), 1 mM D-biotin (Molecular Probes, Eugene, Oregon, USA) in a total volume of 9 μl for 30 min at room temperature; 6 μl of 25pM anti-SP1-DNA conjugate was added to the mixture, and the incubation was continued for 2 h at 20°C. After the incubations, 35 μl of a combined mixture for ligation and real-time PCR amplification/detection was added. Ligation and PCR amplification/detection was performed according to Gustafsdottir *et al*.^31^ Sequences of the primers, probes and the streptavidin–oligonucleotides used in PLA are provided in supplementary table S2.

### Statistical analysis

A χ^2^ test (p>0.05) was used to assess that the genotype distributions of the polymorphisms fulfilled the criteria of Hardy–Weinberg equilibrium. The PLINK software (http://pngu.mgh.harvard.edu/~purcell/plink/) was used to compare the allele counts in cases and controls by Fisher’s exact test and to calculate odds ratios (OR) with 95% confidence intervals. PLINK was also used to perform the sliding window haplotype association analysis. The Haploview v.3.3. software was used to determine linkage disequilibrium (LD) between the polymorphisms. In the family cohort, the genotype data were analysed using the TRANSMIT 2.5.2. computer program package.^32^ Alleles that were included in the analysis were required to be transmitted from a least 20 informative meioses, corresponding to the Var (O-E) value of 5 or more in the TRANSMIT analysis. A combined p value from case–control cohorts and trios were calculated using the analytical expression by Jost, k(1-(ln(k))+(-ln(k)^2^)/2), where k is the product of the p values from the different cohorts (http://www.loujost.com). This formula is an analytical solution of Fischer’s original formula for combining p values.^33^ The ssSNPer tool was used to determine the pair-wise correlation between SNPs in the HapMap data.^34^

## RESULTS

**Figure 1:**
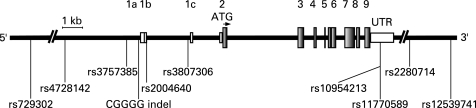
Schematic illustration of the *IRF5* gene with the positions of the analysed polymorphisms. The exons and the 3′-UTR are shown as boxes with the exons labelled 1-9 and the untranslated alternative exons in the 5′-end of *IRF5* labelled as 1a, 1b and 1c. The translation initiation site is indicated by an arrow above the gene. The single nucleotide polymorphism (SNP) rs2004640 is located at the splice junction of alternative exon 1b, where it alters a consensus splice donor site that allows expression of *IRF5* mRNA containing exon 1b.^38^ The SNP rs10954213 located in the 3′untranslated region (UTR) of the gene correlates with an altered length of the *IRF5* 3′UTR and thereby affects the stability of the *IRF5* transcript.^35^ The SNPs rs2280714, located ∼6 kb downstream of the *IRF5* gene, has been reported to be correlated with variation in *IRF5* mRNA expression levels.^35^ ^37^ A 5 bp insertion-deletion polymorphism (CGGGG indel) in the promoter region of *IRF5*, identified by sequencing the *IRF5* gene in Swedish individuals,^35^ was included in our study because it is predicted to alter a binding site for the transcription factor SP1, which could affect the expression of *IRF5*. The SNP rs 12539741 was included because this SNP, and several other SNPs located 3′ of *IRF5* that are in almost full linkage disequilibrium (LD) with it, show very strong association signals with systemic lupus erythematosus.^35^

Ten polymorphisms in the *IRF5* gene on chromosome 7q32 were genotyped in MS patient samples collected in three European countries. The selected polymorphisms include five SNPs and one insertion-deletion polymorphism (indel) in the promoter region or first intron of *IRF5*, two SNPs in the 3′UTR and two SNPs downstream of *IRF5* (fig 1). The polymorphisms were selected because they have previously been shown to be associated with SLE,^25^ ^35^ ^36^ RA^26^ and IBD,^27^ or have been suggested to modulate the expression of *IRF5*.^35^^–^38

The polymorphisms were first genotyped in a Spanish cohort of MS patients (n = 660) and controls (n = 833). Seven of the 10 polymorphisms showed nominally significant signals of association with MS (p<0.05) (table 1).  To replicate this finding, we genotyped the same set of polymorphisms in an independent case–control cohort with 1166 MS patients and 1235 matched controls from Sweden. Two of the SNPs, rs4728142 and rs3807306, and the CGGGG indel polymorphism, showed a nominally significant association (p<0.05) with MS. Each of these three polymorphisms was also associated with MS in the Spanish samples (table 1). In a further attempt to verify these findings from two case–control cohorts of MS patients, we genotyped the same set of polymorphisms in 511 Finnish MS trio families. Using a transmission disequilibrium test, four polymorphisms showed nominally significant association with MS (table 2). Two SNPs, rs4728142 and rs3807306, were nominally significantly associated with MS in all three cohorts (tables 1 and 2).

**Table 1:** Association analysis of *IRF5* polymorphisms with multiple sclerosis in Spanish and Swedish case–control cohorts

Polymorphism	Position (bp)	Location	Major/minor allele*	Risk allele	Risk allele frequency				Risk allele frequency			
					Spanish cohort				Swedish cohort			
**Cases(n = 650)**	**Controls(n = 797)**	**p Value**	**OR (95% CI)**	**Cases(n = 1084)**	**Controls(n = 1182)**	**p Value**	**OR (95% CI)**
rs729302	128356196	Promoter	A/C	A	0.7	0.68	0.22	1.11 (0.94 to 1.30)	0.67	0.66	0.29	1.07 (0.94 to 1.21)
rs4728142	128361203	Promoter	G/A	A	0.52	0.46	**0.0029**	1.25 (1.08 to 1.45)	0.48	0.44	**0.02**	1.15 (1.02 to 1.29)
rs3757385	128364540	Promoter	G/T	G	0.7	0.65	**0.0052**	1.25 (1.07 to 1.47)	0.67	0.66	0.43	1.05 (0.93 to 1.19)
CGGGG indel	128365152	Promoter	del/in	In	0.53	0.48	**0.011**	1.22 (1.05 to 1.41)	0.49	0.45	**0.0093**	1.17 (1.04 to 1.32)
rs2004640	128365537	First intron	T/G	T	0.59	0.53	**0.0011**	1.28 (1.11 to 1.49)	0.55	0.52	0.082	1.11 (0.99 to 1.25)
rs3807306	128367916	First intron	T/G	T	0.57	0.52	**0.0037**	1.25 (1.08 to 1.45)	0.54	0.51	**0.049**	1.12 (1.00 to 1.26)
rs10954213	128376663	3′ UTR	A/G	A	0.69	0.65	**0.031**	1.19 (1.02 to 1.39)	0.67	0.64	0.15	1.10 (0.97 to 1.24)
rs11770589	128376724	3′ UTR	A/G	A	0.57	0.55	0.29	1.08 (0.93 to 1.26)	0.51	0.5	0.49	1.04 (0.93 to 1.17)
rs2280714	128381961	Downstream	T/C	T	0.73	0.68	**0.003**	1.28 (1.09 to 1.51)	0.71	0.7	0.43	1.05 (0.93 to 1.20)
rs12539741	128384041	Downstream	C/T	T	0.11	0.09	0.074	1.27 (0.98 to 1.65)	0.15	0.14	0.10	1.15 (0.97 to 1.36)

*Major and minor alleles in unaffected subjects.

p Values below 0.05 are indicated in bold.

**Table 2:** Association analysis of *IRF5* polymorphisms with multiple sclerosis in the Finnish trios

Polymorphism	Associated allele	Obs-Exp*	p Value
			
rs729302	A	+	**0.047**
rs4728142	A	+	**0.035**
rs3757385	G	+	0.53
CGGGG indel	in	+	0.056
rs2004640	T	+	0.54
rs3807306	T	+	**0.012**
rs10954213	A	+	**0.010**
rs11770589	A	+	0.070
rs2280714	T	+	0.30
rs12539741	T	+	0.74

*Difference between observed and expected transmissions of the associated allele; + denotes increased transmission to affected offspring; p values below 0.05 are indicated in bold.

Information on HLA-DRB1*15 genotype was available from the Swedish cases and controls, and from the Finnish patients (supplementary table S3). In the Swedish dataset most of the association signal was observed in the DR15-negative stratum, whereas in the Finnish dataset the DR2 stratum provided most of the association signal (supplementary table S4). When combining the p values from all cohorts, seven of the 10 polymorphisms exhibited association with MS with p values ranging from 0.0002 to 0.04. The strongest association signals in this combined analysis were observed for the SNP rs4728142 (p = 0.0002) located ∼5 kb upstream of *IRF5*, the CGGGG indel located 64 bp upstream of exon 1a of *IRF5* (p = 0.0005), and the SNP rs3807306 in the first intron of *IRF5* (p = 0.0002) (table 3, fig 1).

**Table 3:** Combined association analysis of *IRF5* polymorphisms with multiple sclerosis in three cohorts

Polymorphism	Spanish c/c*	Swedish c/c*	Finnish trios	Combined p value
	p Value	p Value	p Value	
rs729302	0.22	0.29	**0.047**	0.07
rs4728142	**0.0029**	**0.020**	**0.035**	**0.0002**
rs3757385	**0.0052**	0.43	0.53	**0.04**
CGGGG indel	**0.011**	**0.0093**	0.056	**0.0005**
rs2004640	**0.0011**	0.082	0.54	**0.003**
rs3807306	**0.0037**	**0.049**	**0.012**	**0.0002**
rs10954213	**0.031**	0.15	**0.010**	**0.003**
rs11770589	0.29	0.49	0.070	0.16
rs2280714	**0.0030**	0.43	0.30	**0.02**
rs12539741	0.074	0.10	0.74	0.11

*c/c denotes case–control; p values below 0.05 are indicated in bold.

**Figure 2:**
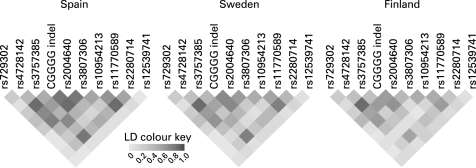
Linkage disequilibrium (LD) structure of the *IRF5* gene. Pairwise r^2^ values are shown for controls or founders in the Spanish, Swedish and Finnish cohorts, respectively. The three most strongly associated markers rs4728142, the CGGGG indel and rs3807306 are in relatively high LD with each other with pair-wise r^2^ values >0.6.

The linkage disequilibrium (LD) pattern of the polymorphisms was similar between the three populations (figure 2), and displayed relatively high LD with pair-wise r^2^ values of 0.61–0.88 for the three associated polymorphisms in the Spanish, Swedish and Finnish unaffected subjects. We performed haplotype association tests in the three populations to investigate whether haplotypes could capture the association signal at a higher significance than the individual polymorphisms, and whether all three populations harbour the same disease associated haplotype(s). The haplotype analysis was performed using a five marker sliding window approach. The associated risk alleles of the polymorphisms were all present on the most common haplotype, which had a frequency of 0.43–0.51 and was the same in all three cohorts (table 4). The association signals were comparable to those in the analysis of individual polymorphisms.

**Table 4:** Sliding window haplotype association analysis:

(1) rs729302; (2) rs4728142; (3) rs3757385; (4) CGGGG indel; (5) rs2004640; (6) rs3807306; (7) rs10954213; (8) rs11770589; (9) rs2280714; (10) rs12539741										Spain			Sweden			Finland			Combined p value	Combined corrected p value‡
(1)	(2)	(3)	(4)	(5)	(6)	(7)	(8)	(9)	(10)											
										F_aff_	F_unaff_	p Value	F_aff_	F_unaff_	p Value	F_obs_*	Obs-Exp†	p Value		
A	A	G	in	T						0.49	0.44	**0.0036**	0.44	0.41	**0.019**	0.41	+	**0.010**	**0.000081**	**0.0032**
C	G	T	del	G						0.17	0.18	0.55	0.17	0.18	0.61	0.20	–	0.42	0.68	NS
A	G	T	del	G						0.13	0.17	**0.0021**	0.15	0.15	0.60	0.14	+	0.47	**0.021**	NS
	A	G	in	T	T					0.51	0.46	**0.0034**	0.47	0.44	**0.019**	0.43	+	**0.028**	**0.00018**	**0.0070**
	G	T	del	G	G					0.28	0.34	**0.0016**	0.28	0.29	0.49	0.26	–	0.51	**0.016**	NS
	G	G	del	G	G					0.10	0.11	0.28	0.12	0.13	0.20	0.10	–	0.29	0.22	NS
		G	in	T	T	A				0.53	0.48	**0.014**	0.49	0.45	**0.0061**	0.45	+	**0.012**	**0.00012**	**0.0046**
		T	del	G	G	G				0.27	0.32	**0.0018**	0.28	0.29	0.53	0.25	–	0.54	**0.019**	NS
		G	del	G	G	A				0.10	0.11	0.29	0.12	0.13	0.28	0.11	–	0.79	0.48	NS
			in	T	T	A	A			0.42	0.39	0.16	0.34	0.32	0.18	0.30	+	0.068	0.052	NS
			del	G	G	G	G			0.27	0.32	**0.0018**	0.28	0.30	0.26	0.25	–	0.52	**0.011**	NS
			ins	T	T	A	G			0.11	0.09	0.068	0.15	0.13	0.068	0.15	+	0.24	**0.034**	NS
				T	T	A	A	T		0.44	0.41	0.13	0.35	0.33	0.11	0.31	+	**0.044**	**0.022**	NS
				G	G	G	G	C		0.27	0.32	**0.0026**	0.28	0.29	0.35	0.25	–	0.42	**0.015**	NS
				G	G	A	A	T		0.11	0.13	0.22	0.13	0.14	0.35	0.13	–	0.56	0.39	NS
					T	A	A	T	C	0.46	0.42	0.076	0.39	0.37	0.19	0.39	+	**0.016**	**0.010**	NS
					G	G	G	C	C	0.27	0.32	**0.0028**	0.28	0.29	0.38	0.25	–	0.44	**0.018**	NS
					G	A	A	T	C	0.11	0.13	0.25	0.13	0.14	0.40	0.13	–	0.59	0.46	NS

*Haplotype frequency in Finnish trios. †Difference between observed and expected transmissions of the associated allele; + denotes increased and – denotes decreased transmission to affected offspring. ‡39 haplotypes were tested and a conservative Bonferroni correction factor of 39 was applied. The three highest frequency haplotypes in each window are shown. p Values are indicated in bold.

**Figure 3:**
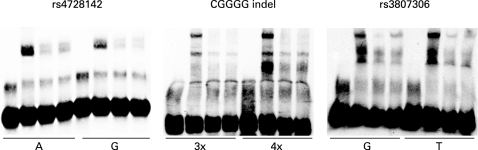
Electrophoretic mobility shift assay images for the three most strongly associated variants of *IRF5*. Reactions loaded in lanes 1–4 (from left to right) for each allele contain: (1) labelled probe only; (2) labelled probe and nuclear extract; (3) labelled probe, nuclear extract and the unlabeled probe, which is added as a competitor in 100-fold excess; (4) labelled probe, nuclear extract, and a 100-fold excess of unlabelled probe for the other allele of the polymorphism added as a cross-competitor.

**Figure 4:**
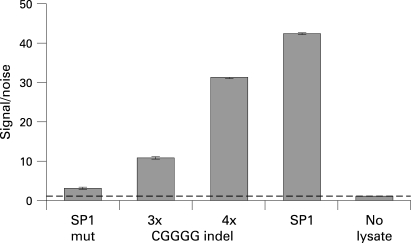
Result from analysis of the SP1 protein–promoter interaction using the proximity ligation assay. The probes included in the assay shown from left to right are: SP1 mut—negative control probe where four nucleotides within the SP1 consensus binding site have been altered; CGGGG indel 3× and 4×—probes with three or four copies of CGGGG; SP1—positive control probe with SP1 consensus binding site; No lysate—control reaction where no nuclear lysate was added to the reaction. The samples were analysed in duplicate and the result presented are mean values of signal/noise, where the signal from the proximity probe pairs in the sample is divided by the signal in the control reaction without cell lysate. The data shown are from a representative experiment repeated three times with similar results. In each individual experiment the signal obtained by the 4× CGGGG indel probe was 2.6±0.3 fold higher than the signal from the 3× CGGGG indel probe.

We used electrophoretic mobility shift assays (EMSA) to test for differential protein binding to the alleles of the three polymorphisms rs4728142, rs3807306 and the CGGGG indel, which displayed association signals with values of p<0.001 in the combined analysis of the three MS cohorts. This analysis revealed a stronger binding of protein to the risk alleles of the SNP rs4728142 (the A allele) and of the CGGGG indel polymorphism (the 4×CGGGG allele) (fig 3). For the CGGGG indel polymorphisms the insertion of one CGGGG repeat in the longer (4×CGGGG) allele is predicted to create an additional and third binding site for the transcription factor SP1, while the shorter (3×CGGGG) allele has two SP1 binding sites (TFSEARCH: http://www.cbrc.jp/research/db/TFSEARCH.html).^39^ By using the proximity ligation assay^31^ ^40^ we confirmed that SP1 protein binds to the alleles of the CGGGG indel, and that an increased amount of SP1 is bound to the 4×CGGGG allele than to the 3×CGGGG allele of the indel polymorphism (fig 4).

## DISCUSSION

Here we describe the association of three polymorphisms in the *IRF5* gene with MS in three independent patient cohorts from Spain, Sweden and Finland. The SNPs rs4728142 and rs3807306 reached nominal significance for association in all cohorts and revealed strong signals of association with MS (p = 0.0002) when the data from all three cohorts was combined. The SNP rs4728142 is located ∼5 kb upstream of the alternative exon 1a of *IRF5*, and rs3807306 is located in the first intron of the gene, 96 bp upstream of alternative exon 1c (fig 1). The SNP rs3807306 was the only *IRF5* polymorphism reported in the recently performed genome-wide association study on MS.^7^ In that study the SNP rs3807306 exhibited association with MS with a TDT p value of 0.014 (https://imsgc.org/), but it did not pass the threshold for inclusion in the confirmatory phase of the study. This study also identified the T allele as the risk allele. It is notable that the initial TDT screening phase of this genome-wide study only had about 6% power to detect each locus at the chosen cut off and with the observed modest risk ratios.^7^ The effect of this low power becomes apparent when looking at the SNPs in IL2RA and IL7RA, which did not pass the initial p value cut off for the TDT, but turned out to be the most strongly associated markers after the replication phase. In our study the CGGGG indel polymorphisms, located 64 nucleotides upstream of the alternative exon 1a of *IRF5*, also showed evidence of association with MS. Although the CGGGG indel did not reach statistical significance independently in all three cohorts, the combined analysis revealed a clear association signal between the CGGGG indel and MS (p = 0.0005), with the longer allele (4×CGGGG) as the risk allele.

The risk alleles of the SNPs rs4728142 and rs3807306 and of the CGGGG indel are present on the same common haplotype in each of the three populations (table 4). In a recent study on IBD we analysed the same set of polymorphisms as in the current study on MS, and found that the same three polymorphisms were associated with IBDs, with the strongest signal of association for the CGGGG indel.^27^ We have recently performed an association study of a comprehensive set of polymorphisms in *IRF5* that were identified by sequencing the introns and exons of *IRF5* in SLE patients.^41^ This analysis identified a set of correlated polymorphisms in *IRF5* that gave strong signals of association with SLE (p<10^-6^), including the SNPs rs4728142, and rs3807306 and the CGGGG indel. In SLE logistic regression analysis conditional only on the CGGGG indel abolished all the other association signals from this set of correlated SNPs. Because the effect size of the polymorphisms are lower in MS than in SLE we cannot distinguish which one of them would be the most likely causal variant in MS. In an earlier association study on RA we analysed five SNPs in *IRF5*, including the SNPs rs729302, rs375385, rs 2004640 and 3807306, but not the CGGGG indel, and found the strongest signal of association with RA for the SNP rs3807306.^26^ Taken together, the association results from the current study on MS and previous studies on IBD, SLE and RA indicate that one or more of these correlated polymorphisms in the promoter and first intron of the *IRF5* gene could be a universal risk factor for chronic inflammatory disorders, but further studies are required to dissect if the mechanism is the same in all of these disorders.

Using EMSA, as a preliminary functional test for the three polymorphisms in *IRF5* that gave the strongest association signals with MS, we observed stronger protein binding to the risk alleles of the SNP rs4728142 (the A allele) and of the CGGGG indel polymorphisms (the 4×CGGGG allele), whereas both alleles of the SNP rs3807306 appear to bind an equal amount of protein. We confirmed experimentally using an antibody against SP1 in the proximity ligation assay^31^ that an increased amount of transcription factor SP1 binds to the risk allele (the 4×CGGGG allele) of the CGGGG indel polymorphism. This result is also supported indirectly by a study in which they demonstrated that SP1 binds to a similar sequence motif in the *IRF1* and *IRF4* genes.^42^ ^43^

It is notable that the SNP rs12539741 located in the 3′-end of the *IRF5* gene does not show any association with MS in our study. In SLE this SNP and two other linked SNPs, the SNPs rs2070197 in the 3′-UTR of *IRF5* and rs10488631 located ∼5 kb downstream of *IRF5*, give particularly strong association signals.^35^ Two recent genome-wide association studies failed to detected an association with RA for the SNP rs10488631 or its proxies^44^ ^45^ and in our study on IBD we did not detect an association with the SNP rs10488631 either.^27^ Thus it appears that in SLE there are two groups of independently associated polymorphisms in the *IRF5* gene region,^46^ whereas in MS, RA and IBD association from only one of these groups is observed. According to data from the HapMap project (www.hapmap.org), the SNP rs10488631 is in complete LD with multiple SNPs located in a 100 kb region downstream of *IRF5*, which also contains the transportin 3 (TNPO3) gene. The polymorphisms in the *IRF5* gene that we found to be associated with MS in our study are not strongly correlated (r^2^ 0.1–0.2) with the SNP rs10488631 or its proxies in the TNPO3 gene,^34^ indicating that it actually is *IRF5*, and not TNPO3, that is primarily responsible for the association with MS that we observe.

Three different European populations were included in the present study, and the observed allele and haplotype frequencies did not show major inter-population differences. The disease predisposing alleles are the same and occur on the same major haplotype in the Spanish, Swedish and Finnish populations. These results indicate that the predisposing alleles are widely distributed in the Caucasian population. The effect sizes of the risk alleles are relatively small, with odds ratios of about 1.2, indicating that large datasets are needed to replicate these findings. The strength of our study is that we used both case–controls and family based association testing. Population stratification artefacts are common in case–control settings, but unlikely in family based studies.^47^ On the other hand, the TDT may be biased by erroneous detection of association to alleles with high frequency in the analysed populations.^48^

MS is a common disease, and most likely caused by interaction between multiple common allelic variants of genes. The association of *IRF5* polymorphisms with MS in the cohorts studied here suggests that *IRF5* is one of these genes that contribute to the disease. Interestingly, in the animal models for MS, experimental autoimmune encephalomyelitis, deletion of the IFN β gene leads to more severe disease,^49^ suggesting that the inherent type I IFN function contributes to the autoimmune disease. Currently, the most common therapy in MS is IFN β, which has been shown to reduce the magnetic resonance imaging activity and relapse rate in MS. This therapeutic effect is consistent with an immunoregulatory role of the type I IFN pathway in MS. The findings from our study add *IRF5* to the short list of genes with confirmed association with MS. Our study also contributes to the evidence that there might be genes or pathways that are common between multiple autoimmune diseases, and that the type I IFN signalling system, to which the *IRF5* gene belongs, is likely to be one of these pathways.
